# The scent of a handshake

**DOI:** 10.7554/eLife.06758

**Published:** 2015-03-03

**Authors:** Gün R Semin, Ana Rita Farias

**Affiliations:** Faculty of Social and Behavioral Sciences, Utrecht University, Utrecht, Netherlands; Koç University, Istanbul, Turkey and ISPA - Instituto Universitário, Lisbon, PortugalG.R.Semin@uu.nl; ISPA - Instituto Universitário, Lisbon, Portugal

**Keywords:** social chemosignaling, Handshaking, Sniffing, pheromones, human

## Abstract

Sniffing our hand after a handshake may allow us to detect chemical signals produced by others.

**Related research article** Frumin I, Perl O, Endevelt-Shapira Y, Eisen A, Eshel N, Heller I, Shemesh M, Ravia A, Sela L, Arzi A, Sobel N. 2015. A social chemosignaling function for human handshaking. *eLife* **4**:e05154. doi: 10.7554/eLife.05154

**Image** Chemosignals can be transferred between individuals when they shake hands

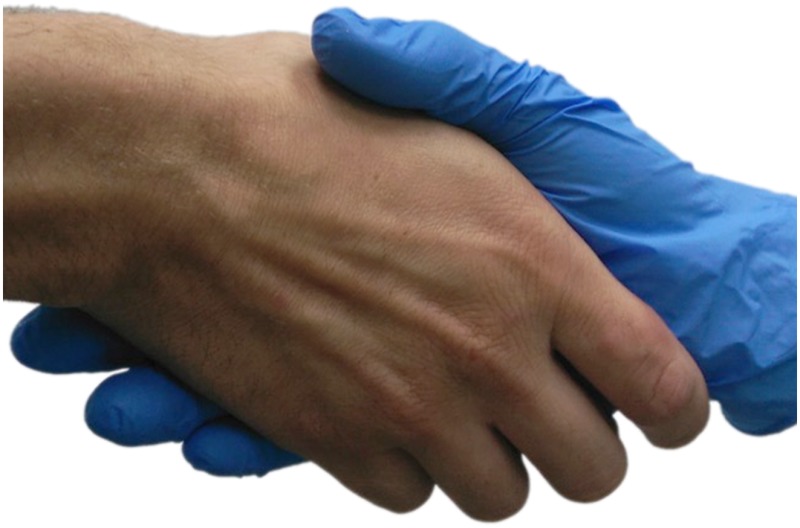

There is growing evidence that human sweat carries a wealth of information that we can detect. Studies have revealed that human sweat can indicate the gender and age of a person (Penn et al., 2007; Mitro et al., 2012). Sweat can also act as a signal of emotional states such as fear or happiness (e.g., Prehn et al., 2006; Mujica-Parodi et al., 2009; Zhou and Chen, 2009; de Groot et al., 2015). For instance, if someone sniffs the sweat produced by someone else while they were experiencing fear, it can cause fearful emotions in the first person (de Groot et al., 2012).

The chemicals we can detect in sweat are known as chemosignals. Research on social chemosignaling has mostly relied on experiments that expose volunteers to sweat. Fascinating as this research may be, an important question has not been asked before: do humans actually make any attempt to smell the odors of others? Other mammals are known to actively engage in such ‘olfactory sampling’ but there was no evidence of anything resembling these behaviors in humans. Now, in *eLife*, Noam Sobel and co-workers at the Weizmann Institute of Science—including Idan Frumin as first author—present a remarkably ingenious and carefully designed series of studies revealing that handshaking is very likely to serve this purpose in humans (Frumin et al., 2015).

To start with, Sobel, Frumin and co-workers showed that it is possible for chemicals involved in social chemosignaling to be transferred between people during a handshake. This opened the door for the critical experiment in which volunteers—who believed they were waiting to take part in a research study—were greeted by an experimenter who entered the room three minutes after the volunteers were seated. During the 20 second greeting, the experimenter either shook the right hand of the volunteer, or they did not shake hands at all. The researchers then analyzed the hand movements of the volunteers for 80 seconds before and after the greeting via hidden video recordings.

Frumin et al. found that after shaking hands with an experimenter of the same gender, both male and female volunteers held their right hand close to their face for longer than the volunteers who did not shake hands. However, the volunteers who shook hands with someone of the opposite gender spent more time with their left (i.e. non-shaking) hand near their face. Indeed, the analysis also revealed that their hands contacted their face in the immediate vicinity of their nose (Figure 1). Frumin et al. also examined the airflow in the nose before and after a handshake. They found that when a volunteer held their hand close to their nose, the airflow in the nose was more than twice the level during normal breathing, which suggests that the volunteers were indeed sniffing their hands.

**Figure 1.**
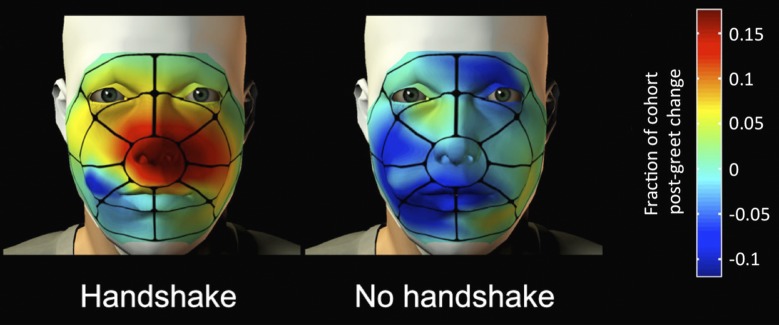
After a handshake, the volunteers spent more time with their hand held in the vicinity of the nose. The spatial distribution of the contact between the right hand and the face following a greeting with a handshake, or without a handshake. Red indicates the areas where hand touching increased in many of the volunteers after the greeting; dark blue indicates areas where it decreased. Turquoise indicates areas where little or no change in hand touching was observed.

To demonstrate that the different sniffing patterns observed for same gender and different gender handshakes were indeed due to the presence of different odors, Frumin et al. carried out a final experiment with female volunteers and female experimenters in which they tainted the experimenters with one of three odors: a chemical that is believed to be a male social chemosignal; a chemical that is believed to be a female social chemosignal; and a commercial unisex perfume.

Frumin et al. predicted that the gender-specific odors should alter the right and left hand sniffing patterns observed in the main study. Indeed, the volunteers that shook hands with an experimenter tainted with the female chemosignal sniffed their right hand for a shorter time, and sniffed their left hand for longer, compared to those who shook hands with untainted or perfumed experimenters. Oddly, the male chemosignal had the same effect on the behavior of the volunteers as the female chemosignal, although this effect was weaker. These results suggest that different odors can alter hand-sniffing patterns after a handshake.

This carefully packaged set of studies opens a new door in chemosignaling research by showing that humans do engage in olfactory sampling. This behavior probably extends to other rituals we engage in when we see a friend or colleague such as hugging, embracing, holding each other and kissing each other's cheeks.

The findings pose many new research questions. For example, what is the information that is conveyed by the chemosignals that are being sampled? How do such chemosignals affect human behavior, beyond the hand sniffing behaviors demonstrated in this research? Why do same gender and opposite gender handshakes produce different patterns of hand sniffing? Could these patterns be driven by cultural factors?

Conducting observational studies of greetings between people in more natural settings—at conferences or other social events for instance—could reveal how the environmental context affects odor sampling in humans. The elegance of introducing a new perspective, as Sobel, Frumin and co-workers have done in this study, is that it not only opens our eyes to a previously ignored phenomenon, but also stimulates new research questions.
